# Urban Chinese Community-Dwelling Older Adults’ Expectations Regarding the Delivery of Integrated Care Through Case Managers: Protocol for a Mixed Methods Study

**DOI:** 10.2196/71394

**Published:** 2025-11-07

**Authors:** Yuanyuan Zhao, Boon-How Chew, Feng Zhou, Yuehua Tu, Hua Zhu

**Affiliations:** 1 School of Smart Health and Wellness (Health Medical College) Zhejiang Dongfang Polytechnic Wenzhou City, Zhejiang Province China; 2 Department of Family Medicine, Faculty of Medicine & Health Sciences Universiti Putra Malaysia Serdang Malaysia; 3 School of Engineering Guangzhou College of Technology and Business Guangzhou China; 4 School of International Education Jiangxi Science and Technology Normal University Nanchang China; 5 School of Smart Health and Wellness (Health Medical College) Zhejiang Dongfang Polytechnic Wenzhou China

**Keywords:** aging, chronic diseases, case management, person-centered care, qualitative research, quantitative research

## Abstract

**Background:**

The rising burden of chronic diseases among older adults in China underscores the urgent need for an integrated and efficient health care system. Existing services are often fragmented, lacking coordination and accessibility, particularly for urban community-dwelling older adults with multiple comorbidities. There is a critical demand for person-centered integrated care that enables resource sharing, expert guidance, and the safe, continuous management of chronic conditions.

**Objective:**

This study aims to extend the theoretical understanding of case-managed integrated care informed by the Program of Research to Integrate the Services for the Maintenance of Autonomy (PRISMA) model within urban Chinese communities. It will examine older adults’ expectations for case-managed integrated care, develop and validate a measurement instrument to quantify these expectations, and build an evidence-based framework to adapt the PRISMA model to the Chinese context.

**Methods:**

An exploratory sequential mixed methods design will be used across 3 phases. Phase I involves qualitative interviews with community-dwelling older adults and other stakeholders to explore unmet health care needs and perceptions of case-managed integrated care. Phase II entails the development and psychometric validation of a questionnaire informed by the findings from phase I. Phase III involves a large-scale cross-sectional survey in Wenzhou, Taiyuan, and Hainan to quantify expectations for case-managed integrated care and identify associated sociodemographic factors. A data integration approach will be used to synthesize both qualitative and quantitative findings, providing a comprehensive understanding of expectations regarding the proposed integrated care.

**Results:**

Data collection is scheduled to begin in December 2025, with the study expected to last 24 months. Ethics approval has been obtained from the Institutional Ethics Committee.

**Conclusions:**

This study seeks to evaluate the feasibility of culturally adapting the PRISMA model for case-managed integrated care to address critical gaps in existing health care service delivery. The findings are expected to inform policy formulation, guide the implementation of integrated community care strategies, and ultimately improve health outcomes and quality of life for older adults in urban China.

**Trial Registration:**

OSF Registries 825ah; https://osf.io/825ah

**International Registered Report Identifier (IRRID):**

PRR1-10.2196/71394

## Introduction

### Background

The National Bureau of Statistics reported that by the end of 2023, China’s aging population (60 years or older) surpassed 290 million, representing approximately 21.1% of the total population [1]. More than half of this demographic faces high morbidity rates, with highly prevalent conditions such as type 2 diabetes, chronic obstructive pulmonary disease, and hypertension [2,3]. Furthermore, approximately 96 million older adults experience varying degrees of disabilities [4], creating significant demands for medical services and nursing care, including home visits for medication, health monitoring, health care education, and psychological counseling [5-7]. The growing burden of chronic disease highlights the urgent need for more coordinated and effective older adult care systems [8].

The majority of older adults prefer “aging in place”, a choice deeply rooted in the traditional Chinese value of “filial piety” [9,10]. The “9073” care model serves as the primary framework for older adult care in China, wherein 90% of older adults rely on home-based care with community support, 7% reside in community-based institutions or daycare centers, and 3% live in long-term care facilities [11]. However, this model becomes increasingly critical when older adults encounter difficulties performing basic activities of daily living (ADL) due to frailty [12]. Notably, less than 10% of home-based older adults with varying levels of care dependency have received adequate community services, reflecting a significant gap between care provision and demands [13].

To address these challenges, China launched the Integrated Care for Older People program (ICOPE-CHINA) in 2020, following the World Health Organization’s ICOPE framework. While this program integrates health care services, daily living assistance, and social services, many older adults remain unaware of available resources due to limited information and low health literacy [14-16]. Developing accessible and navigable community-based care is therefore a pressing priority [17].

### Literature Review

Globally, several models of integrated care have emerged in response to aging populations and the growing burden of chronic disease. These include the chronic care model (CCM), the Kaiser Permanente model, and the Program of Research to Integrate the Services for the Maintenance of Autonomy (PRISMA). The CCM focuses on clinical settings, self-management support, and proactive care, but it requires extensive system-level transformation, making large-scale implementation difficult in China due to uneven development and constrained resources [18,19]. The Kaiser Permanente model, which integrates funding, information technologies, and payment mechanisms, has proven effective within vertically integrated private systems [20]. However, in the Chinese health care system, it may be more feasible to strengthen collaboration across medical specialties, partner with community organizations and enterprises, and pursue reforms at the county or community levels [21,22]. The PRISMA model was explicitly designed for publicly funded universal health care systems and emphasizes collaboration among otherwise independent service providers [23]. Rather than requiring structural mergers, it establishes mechanisms for linkage and collaboration across health care providers, social service agencies, and community organizations.

The current older adult care system in China comprises community medical centers, community elderly care facilities (CECFs), and voluntary agencies, all of which have evolved in response to government policies, financing mechanisms, consumer demands, and market dynamics [24]. Particularly, CECFs operate under government supervision, offering daycare and temporary services designed to alleviate the burdens of family caregivers. These services include basic nursing care, health case management, household support, rehabilitation, and counseling [25]. In several pilot cities, community-based initiatives have been launched, beginning with the deployment of social workers and the establishment of CECFs to support older adults across multiple ecological levels [26-29]. From a theoretical perspective grounded in the Social Ecological Model [30], existing models differ in their suitability for China’s multilevel, publicly dominated care structure [31]. To assess the models’ contextual fit, the IMPACT Framework is adapted to guide and compare their matching across 6 dimensions (Multimedia Appendix 1 [18-34]) [35]. At the implementation and policy levels, the CCM’s emphasis on individual self-management and clinical integration is not aligned with China’s fragmented, community-based care system [18,19]. The Kaiser Permanente model, though effective in private integrated systems, is less adaptable to China’s fragmented, public-sector-dominated context [21,22,31].

In contrast, the key components of the PRISMA model, including needs assessment, case management, and coordinated referrals, address the major weaknesses observed in China’s current community-based care system. These components directly respond to the challenges faced by Chinese urban community-dwelling older adults, such as limited awareness of available services, fragmented resource use, and insufficient continuity of care [32,33,36]. In particular, the central role of case managers in PRISMA provides a feasible mechanism to guide older adults and their families through complex service pathways, thereby ensuring equity in access and promoting better integration of community resources [37-47]. The detailed functions of case managers are summarized in Table 1.

**Table 1:** The roles and functions of case managers [48].

Main function	Working scope
Advocacy	Advocates for patients’ rights and interests, addressing systemic barriers to care.
Care coordination	Facilitates communication and coordination among patients, caregivers, and health care providers, ensuring seamless care transitions.
Case monitoring and patient needs assessment	Assesses patient needs, sets care goals, and monitors care plans for effectiveness.
Community engagement	Collaborates with community groups to disseminate knowledge and raise funds.
Education	Educates patients, caregivers, and health care professionals on treatments, complications, and financial support options.
Administration and research activities	Mobilizes resources, ensures cost-effectiveness, and supports health care research.
Psychosocial support	Provides emotional support, grief counseling, and crisis intervention.
Navigation of services	Connects patients and caregivers to relevant health care services and resources.
Reduction of barriers	Minimizes obstacles to accessing timely services and treatments.

Case manager certification has been included in China’s National Occupational Classification since 2005, reflecting formal recognition of this role, and the number of graduates trained in care management continues to grow [49]. Although a few studies have piloted case management interventions in China, showing potential in reducing emergency attendance, hospital readmission, health care costs, and enhancing patient satisfaction [50,51], these studies primarily focus on hospital-based applications, with limited attention given to community older adult care, where most older adults reside [52,53]. Beyond this limited focus, fragmented communication among stakeholders further undermines continuity of care in community settings [7]. A previous study found that community residents aged 18 years or older with chronic diseases placed high demands on service continuity, convenience, coordination, responsiveness, and upward information sharing, where case managers could play a critical role [54]. However, limited research has focused on the multifaceted needs of older adults and their centrality to person-centered care. Critically, there is no validated measurement tool to assess older adults’ expectations of case-managed integrated care in the Chinese context. These gaps hinder both policy adaptation and practical implementation of integrated care at the community level.

### Study Objectives

This study aims to extend the theoretical understanding of case-managed integrated care informed by the PRISMA framework within urban communities under a non-Western, publicly funded health care system. It will also contribute to the literature by elucidating how culturally embedded expectations shape the acceptability and potential adaptation of case-managed integrated care in China.

We hypothesize that the urban Chinese community-dwelling older adults with complex health needs and limited access to coordinated services will demonstrate high acceptability of the PRISMA model and express strong expectations for case-managed integrated care. To address this hypothesis, the study primarily pursues the following objectives: (1) investigate the expectations of urban Chinese community-dwelling older adults for case-managed integrated care; (2) develop and validate a measurement instrument to quantify their expectations; and (3) formulate an evidence-based framework for adapting the PRISMA model to the Chinese context. In the study process, we will also explore unmet health care needs, assess the acceptability of a PRISMA-informed case management model, and analyze expectation levels together with associated sociodemographic factors.

## Methods

### Overview

**Figure 1:**
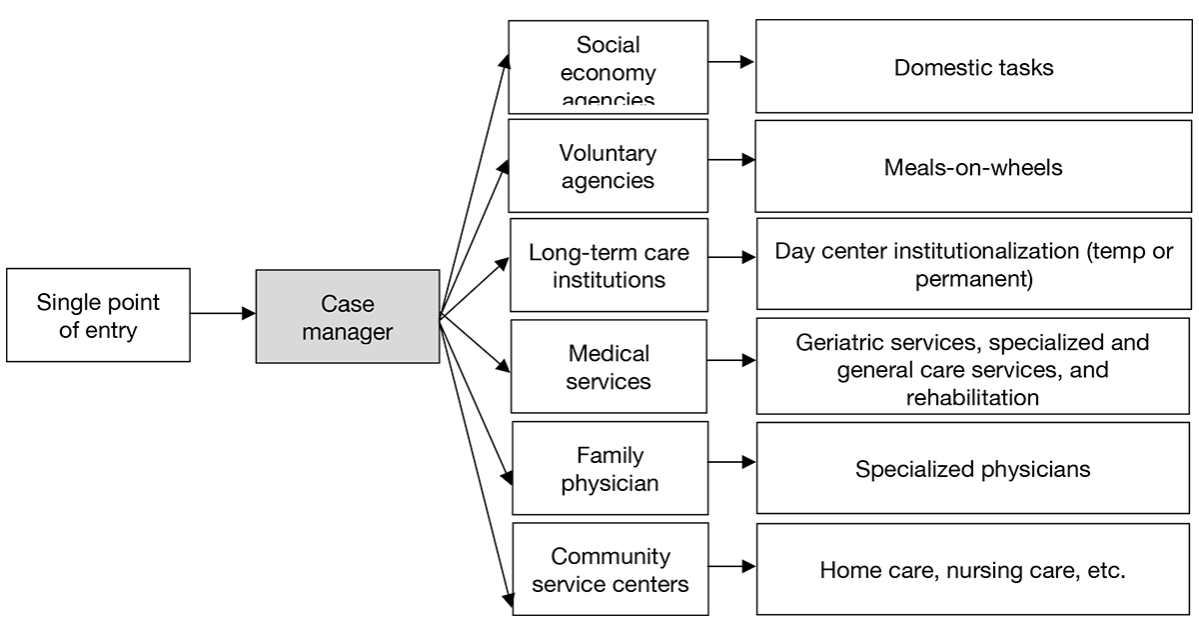
The Program of Research to Integrate the Services for the Maintenance of Autonomy (PRISMA) model of integrated care.

An exploratory sequential mixed methods approach will be used to address the study objectives, ensuring both depth of exploration and measurement validity. The Checklist of Guidelines for Conducting and Reporting Mixed Research for Counselor Researchers will be used to guide the study process and reporting results (Multimedia Appendix 2) [55]. Guided by the PRISMA model (Figure 1), the design consists of 3 interconnected phases [23]. In phase I, a qualitative inquiry will involve in-depth interviews and focus groups to explore urban community-dwelling older adults’ expectations for case-managed integrated care to address objective 1. Thematic analysis will identify key domains of expectations and unmet health care needs among urban community-dwelling older adults and their family members. The acceptability of the PRISMA-informed case management model will be assessed. Phase II will focus on developing and validating a questionnaire to address objective 2. Phase III will use a quantitative survey conducted in 3 selected cities representing the eastern, north-central, and southern regions of China, which are culturally and geographically diverse [56]. The validated instrument will be used to examine expectation levels and associated sociodemographic factors. These empirical data will inform the formulation of an evidence-based framework tailored to the Chinese context and aimed at facilitating the real-world implementation of case-managed integrated care for urban older adults, thereby addressing objective 3. A data integration approach will be used, where themes and codes identified in the qualitative inquiry will inform the development of questionnaire items. Quantitative results will then be analyzed in relation to the initial qualitative findings to confirm, refine, or expand the emerging patterns, ensuring methodological rigor and enhancing integration validity [57].

### Ethical Considerations

Ethics approval for this study was obtained from Zhejiang Dongfang Polytechnic, China (approval number GZSQ202408120010). All participants will receive an information sheet explaining the study purpose, procedures, potential risks, and their right to withdraw at any time without penalty. Written informed consent will be obtained from all participants before interviews or surveys. No identifiable personal information will be included in the future publication.

### Phase I: A Qualitative Study

#### Overview

During phase I, a qualitative case study will be conducted using purposive sampling to recruit participants [58]. The study aims to investigate unmet health care needs of the older adults in the existing urban health care services or systems, assess the acceptability of the PRISMA model, and explore practical requirements for integrated care facilitated by case managers among urban Chinese community-dwelling older adults. Participants will be introduced to the PRISMA model and provided with a semistructured interview guide in simplified Chinese, which includes probing questions. Discussions of health care needs will consider the current health care landscape in China [59]. Three trained investigators will conduct interviews on-site.

#### Settings

The study will be carried out in community medical centers or CECFs located in Wenzhou, Zhejiang Province, China, which play a pivotal role in older adult care by providing essential medical treatment, disease prevention, rehabilitation, and care services to older adults [60,61]. Wenzhou is characterized by a substantial aging population, with older adults accounting for 19.3% of its total population, positioning the city on the trajectory toward a super-aging society [62]. Moreover, Wenzhou has planned a program where community social workers coordinate social services and health care assistance for older adults living in the community, assigning one social worker for every 500 older adults [63]. Participants will be selected from these settings based on their engagement with health care services [64].

#### Participants

Adults aged 60 years or older living in urban communities will be included in the study. The purposive sampling approach will guide participant selection, aiming to capture diverse sociodemographic factors. Inclusion criteria include (1) having 2 or more physician-diagnosed chronic conditions such as ischemic heart disease, stroke, chronic obstructive pulmonary disease, or type 2 diabetes, as documented in their medical records [65]; and (2) self-report visits to community medical centers or hospitals more than 12 times per year for chronic disease management, verified through medical records [66]. To enhance data richness, perspectives from the adult children of eligible older adults, care administrators, and health care professionals within communities will also be invited to the study. They will comprise approximately 50% of the interviews and will be proportionally distributed across the 3 study cities to ensure regional balance and facilitate integration into the analysis and subsequent questionnaire development. Exclusion criteria include unwillingness to participate or having communication difficulties caused by language barriers, cognitive impairments such as dementia, or swallowing disorders like dysphagia.

#### Sampling Process

To achieve demographic diversity and minimize selection bias, recruitment will take place across multiple urban communities with diverse socioeconomic profiles. Investigators will engage both high- and low-income districts to ensure a diverse background of participants. Multiple strategies will be used to approach participants, including direct contact, staff referrals, and distribution of flyers. Demographic factors will be monitored during recruitment to guide further purposive selection. Detailed study information, including the PRISMA model and interview procedures, will be explained to participants.

#### Data Collection

Data will be collected through in-depth interviews and focus groups conducted either face-to-face or online by WeChat (the most popular Chinese app). Each focus group will include up to 4 participants. Informed consent will be obtained, and the PRISMA model, along with the role of case managers, will be explained in clear and accessible language. Semistructured interview guides with probing questions will be used to elicit detailed responses. Interviews will be recorded in video or audio format. Prior to the main investigation, a pilot interview with 2-3 participants will be conducted to refine interview questions. Sociodemographic factors including age, education level, income, presence of chronic diseases, and living arrangements, will be collected. Additionally, the Chinese version of the Barthel Index (BI) will assess participants’ ADL. A total of 30 participants are planned for recruitment, with the final sample size determined by data saturation. A sample of open-ended questions and probes is included in Multimedia Appendix 3.

#### Qualitative Data Analysis

Raw data will be managed using NVIVO (version 11; QSR International Pty Ltd) or ATLAS.ti (version 8; ATLAS.ti Scientific Software Development GmbH). Interviews will be transcribed verbatim by trained investigators, with a second investigator conducting an audit for accuracy. Thematic analysis will combine both inductive and deductive approaches, guided by the PRISMA model and the framework method. The latter consists of 6 steps: transcript review, coding, team discussions, theme comparison, theme definition, and report writing [67].

### Phase II: Questionnaire Development and Validation

#### Overview

The objective of phase II is to develop and validate a questionnaire based on the PRISMA model and the findings from phase I, guided by an item adoption quality control method [68]. This measurement instrument aims to assess older adults’ expectations for case-managed integrated care, capturing both general and specific expectations.

#### Questionnaire Design and Measurement

The questionnaire will consist of 2 main sections. Section 1 will include the sociodemographic factors such as age, income, presence of chronic diseases, assessment of ADL, and one item related to the acceptability of case-managed integrated care. Section 2 will be informed by the qualitative outcomes of phase I related to expectations, with variables derived from coded data and scales aligned with identified themes. Questionnaire items will be constructed using quotations from the qualitative study, guided by the PRISMA model and emerging codes from phase I. A 5-point Likert scale will be used to quantify the level of expectations. Responses will be categorized into 3 levels: low, medium, and high. This categorization can be applied at both the item and construct levels, depending on the properties of the scale. The final questionnaire is expected to include fewer than 50 items related to expectations, with an estimated completion time of approximately 30 minutes. To minimize potential respondent fatigue, the questionnaire will undergo pretesting with older adults to assess burden and ensure acceptability.

#### Questionnaire Validity and Reliability

The face and content validity of the questionnaire items will be evaluated by a panel of at least 6 experts, such as nurses, geriatricians, statisticians, health care case managers, and social workers, as well as older adults. Feedback from the panel will be used to calculate the Content Validity Index for each item [69]. The relevance, comprehensibility, and comprehensiveness of the items will be assessed using the consensus-based standards for the selection of health status measurement instruments checklist [70]. Exploratory factor analysis will be used to examine the underlying factor structure, retaining factors with an eigenvalue greater than 1. Items with factor loadings below 0.40 or with significant cross-loadings with another item will be excluded [71]. Structural Equation Modeling will then be used to assess model fit, ensuring alignment with the hypothesized factor structure. Internal consistency will be measured using Cronbach α, with a threshold of 0.70 or higher indicating acceptable reliability [72]. Additionally, intraclass correlation coefficients will be used to assess test-retest reliability among participants from distinct geographic regions. A random sample of at least 50 participants (16-17 participants at each site) will complete the questionnaire twice, with a one-month interval between administrations [70]. An intraclass correlation coefficient value between 0.50 and 0.75 indicates moderate reliability, while a value of 0.75 or higher signifies good reliability [73]. Beaton’s cross-cultural adaptation framework will be applied to ensure the PRISMA model is culturally appropriate for the Chinese context. This process will include a forward-backward translation procedure conducted by 2 independent bilingual translators, followed by a review from an expert panel consisting of 3-6 professionals, and cognitive interviews with 10 Chinese older adults [74]. In addition, pretesting with 30-40 participants will be conducted to assess clarity, cultural relevance, and comprehension, or as part of the reliability and validity testing in Phase III [75]. Items judged as unclear or culturally inappropriate for ≥20% of participants will be revised or excluded [76].

### Phase III: A Quantitative Study Through a Survey

#### Overview

In Phase III, a survey will be conducted to examine the expectations of case-managed integrated care among urban Chinese community-dwelling older adults, along with their associated sociodemographic factors, using the validated questionnaire developed in Phase II. The questionnaire will be administered in Chinese via a web-based platform wjx.cn or distributed as printed hard copies along with consent forms. A triangulation matrix will align key research questions with both qualitative and quantitative data sources, supporting convergence and complementarity across datasets. In the final phase, the merged data will be analyzed to provide a more comprehensive understanding of complex phenomena, test hypotheses, and deepen insights [77]. Based on the results, an evidence-based framework will be developed to bridge the gap between the global model and local implementation.

#### Settings

China’s population distribution and economic development vary significantly across regions, with a general decline in economic indicators from east to west and north to south, reflecting substantial sociodemographic diversity [78]. To capture this diversity, the survey will be conducted in 3 major cities: Wenzhou, Taiyuan, and Hainan Island, each representing distinct geographic, economic, and demographic profiles. Wenzhou, located along China’s eastern coast, is an economically developed area characterized by a thriving private economy and a significant aging population who predominantly prefer to age in place within their communities [79,80]. Taiyuan, the capital of Shanxi province in northern-central China, has an older adult population exceeding 21.9%, indicating a super-aged society. However, average income levels are slightly below the national level, reflecting a less economically developed context [81,82]. Hainan, China’s southernmost island province, is experiencing rapid aging, with older adults comprising 15.5% of its population. The province is notable for its diverse aging demographic, which includes seasonal migrants from 27 provinces, providing a unique representation of mobility patterns and demographic heterogeneity among older adults [83,84]. The inclusion of these 3 locations ensures the study’s findings will be generalizable across varied socioeconomic, cultural, and geographic contexts, thereby enhancing the representativeness of the aging population in China.

#### Participants and Sample Size Estimation

Participant selection will focus on older adults aligned with the inclusion criteria in phase I, including (1) age of 60 years and older, (2) living independently in the community, (3) having 2 or more chronic conditions, and (4) visiting community medical centers or hospitals more than 12 times per year for chronic disease management. Exclusion criteria are unwillingness to participate, language barriers, or cognitive impairments. This study primarily aims to estimate the proportion of older adults expecting integrated care services and to examine sociodemographic factors associated with different expectation levels. Sample size estimation was conducted using multiple scenarios. Assuming a 95% CI level (Z=1.96) and a margin of error (e=0.05), the required sample size varies according to the expected proportion (P) of older adults expressing high expectations for integrated care. If P is 0.5 (indicating maximum variability), the required sample size is 385. Additionally, to allow for subgroup analysis (eg, by income, education, or ADLs) and to detect a medium effect size (Cohen w=0.3) [85] with 80% power using chi-square tests across 3 groups (low, medium, and high expectation levels), the minimum required sample size is approximately 108 per group, totaling 324 participants. To ensure representativeness and account for a 20% nonresponse rate, the target sample size will be at least 480 participants (160 in each city).

#### Sampling Process

A stratified random sampling method will ensure representation across the diverse aging population. Enumerators (2 in each city) will identify 4 communities in each city where older adults comprise over 21% of the population. Two of the communities will then be randomly selected using an online random number generator, such as Random.org, or the RAND function in Excel. Recruitment will occur through posters, flyers, announcements, and outreach via digital platforms within the community, such as medical centers or CECFs. Participants will provide demographic information, which will be used to stratify the sample by age and chronic disease status before random selection within each stratum.

#### Data Collection Process

At each site, one investigator and one research assistant will oversee data collection. On-site investigators will screen older adults against the inclusion criteria and provide eligible participants with detailed study information. Informed consent will be obtained from participants. To facilitate follow-up and support test-retest reliability, participants may optionally provide telephone numbers. All collected data will be entered into a database and subjected to a double-checking process by investigators and research assistants to ensure completeness.

#### Measurement

At the beginning of the questionnaire, a concise explanatory note in Chinese will be provided to describe the modified PRISMA model. This note outlines the concept of integrated care, managed through a single-entry point by a case manager, to ensure participants understand the key concept before proceeding. Verbal explanations will be provided upon request. To ensure ethical rigor, additional safeguards will be applied for participants with potential cognitive impairments, including the use of simplified consent materials, a brief assessment of decisional capacity prior to enrollment, and involvement of family members to support understanding. Investigators will be trained to identify signs of distress or reduced comprehension, ensuring the voluntary and informed participation of all respondents.

#### Sociodemographic Factors

Age and the frequency of hospitalization in the past year will be collected as numerical variables. Other variables, including gender (male or female), health status (healthy, living with one chronic disease, and living with 2 or more chronic diseases), monthly income (<US $375, US $375-749, >US $749), education level (no formal education, elementary school, middle school, high school, bachelor degree or higher), occupation (employed, unemployed, and retired), insurance (no insurance, Urban Employee Basic Medical Insurance, Urban Resident Basic Medical Insurance, and New Rural Cooperative Medical Insurance), number of children (no child, 1 child, 2 children, and 3 or more children), and living arrangements (alone, with a partner, with children, with a partner and children, and with others such as a housemaid) will be categorical variables.

### BI

The BI scale will assess participants’ self-care abilities across 10 domains, including bowel and bladder control, feeding, bathing, dressing, and walking. Each domain will be scored on a scale of 0-15 points, reflecting the levels of assistance required [86]. The validated Chinese version of the BI scale will be used [87,88], with scoring categories as follows: 100-91 (complete independence), 90-61 (slight dependence), 60-21 (moderate dependence), and ≤20 (severe dependence) [89].

### Expectation Section

The final questionnaire will categorize variables into individual items and grouped constructs to measure participants’ overall and specific expectations of case-managed integrated care. A 5-point Likert scale (1=low expectation, 5=high expectation) will be used.

### Data Analysis for the Quantitative Study

Quantitative data will be analyzed using SPSS Statistics (version 26; IBM Corp). Data from digital questionnaires will be exported to SPSS, while hard-copy data will be manually entered into the dataset. Mean expectation scores will be analyzed either as a continuous variable or divided into tertiles (lowest, middle, and highest). Chi-square tests will examine associations between sociodemographic factors and expectation levels. Statistical significance will be set at *P*<.05, with 95% CIs. Multiple logistic regression models will be used to evaluate the association between independent variables and expectation levels, with the lowest subgroup serving as the reference category. Variables with a *P* value <.20 in univariable regression will be included in the multivariable model. Multicollinearity will be assessed using a tolerance threshold of <0.4 or a variance inflation factor of ≥2.5. Model adequacy will be evaluated through Q-Q plots for normality, residual plots for linearity, and tests of homogeneity. For missing data, descriptive analyses will be conducted to assess the extent and patterns of missingness. When the proportion of missing data is low, complete-case analysis will be applied. In cases of substantial missingness, multiple imputation will be used if feasible; otherwise, simple imputation methods such as mean substitution for continuous variables and mode substitution for categorical variables will be considered [90]. Multilevel logistic regression will also be considered to assess and account for the possible geographic clustering and contextual variation across regions. Results from both statistical strategies above will be compared and interpreted accordingly. These modeling strategies aim to ensure robust and reliable findings that inform evidence-based health care frameworks for older adults in Chinese communities.

## Results

This study was funded in February 2025 and received ethical approval from the Institutional Ethics Committee (approval number GZSQ202408120010). Data collection is scheduled to begin in December 2025 and is expected to be completed by December 2027. As of manuscript submission, participant recruitment has not yet commenced, and data analysis has therefore not begun. The primary results are expected to be available in late 2027.

## Discussion

This study aims to explore the expectations of case-managed integrated care among older adults in Chinese communities. The PRISMA model, emphasizing a single-entry point, case management, and multidisciplinary collaboration, provides a robust theoretical foundation to guide the study design and interpret outcomes across 3 phases. By contextualizing this model within a non-Western, community-based care system, the study seeks to bridge the gap between evidence and practice and to examine how culturally embedded expectations influence the acceptability and adaptation of case-managed integrated care in China. Additionally, the study aims to identify effective pathways to support the implementation of integrated care initiatives, such as the ICOPE-CHINA program.

The acceptability and implementation of case management in China must be understood within the framework of traditional cultural values, such as filial piety and family-centered care. Older adults have traditionally relied on their adult children for care and support [91]. However, demographic and social changes, particularly the one-child policy, have reshaped family structures, resulting in an increasing number of older adults living alone or without immediate family support [92]. Today, the living arrangements of older adults are increasingly influenced by social support and health conditions, making co-residence with children less common or feasible [93,94]. As a result, the role of the family is shifting from direct caregiving to supervisory and coordinating functions, creating opportunities for community-based integrated care and more effective resource navigation [95].

The PRISMA model’s core tenets, such as individual autonomy, personalized care planning, and self-directed decision-making, often reflect individualistic care paradigms that may not align with collectivist norms in China, where health care decisions are often made within family units [96,97]. This cultural incongruity may affect the model’s acceptability and implementation in Chinese communities. To address this, the expectations of other key stakeholders will be investigated in phase I, particularly the adult children who often play a central role in care-related decisions, to assess whether and how the PRISMA model’s individualistic foundations can be adapted to align with collectivist norms, including shared decision-making, filial responsibility, and the involvement of extended family and community networks.

Unlike existing instruments used in China, the questionnaire developed in phase II is theoretically grounded, empirically testable, and specifically tailored to assess the expectations of urban community-dwelling older adults regarding case-managed integrated care. For example, the Patient-Reported Experience Measures mainly capture hospital-based experiences and may overlook patients’ perspectives on continuity and coordination across community and social care settings [98,99]. The Chinese version of the Rainbow Model of Integrated Care Measurement Tool evaluates the effectiveness of integrated care from system-level or provider perspectives [100]. These instruments also lack alignment with the micro-level expectations of older adults. Similarly, the Chinese Patient-Centered Integrated Care scale addresses patient-centeredness but does not emphasize aging populations or the role of case management [54]. In line with the IMPACT framework, particularly the matching dimension, this study ensures theoretical coherence by aligning the PRISMA model’s macro-level foundation with meso- and micro-level constructs derived from qualitative findings [35]. This alignment enhances both conceptual clarity and practical relevance. The construct development process also reflects parsimony by focusing on core expectation dimensions, with empirical testability supported through pilot testing and expert validation. Furthermore, the questionnaire is culturally adapted to China’s evolving community-based older adult care system, thereby enhancing its contextual fit. Collectively, these features position the instrument as a novel and relevant contribution to the evaluation and model design of integrated care for older adults in similar settings.

A key strength of this study lies in its adoption of a mixed methods design, integrating qualitative and quantitative approaches to comprehensively address complex research questions and ensure triangulation of findings, guided by a validated checklist [55,57]. This integrated, multiphase approach ensures that qualitative insights inform the development of a context-specific measurement instrument, validated through rigorous quantitative analysis.

Phase I will gather qualitative data through in-depth interviews with Chinese older adults to explore unmet health care needs, perspectives on the PRISMA model, and expectations for a case manager system. A purposive sampling approach will ensure demographic diversity among participants. Reflexive practices, including audio recordings, detailed transcriptions, involvement of multiple investigators, and peer debriefing, will mitigate potential interpretive bias, enhance credibility, and ensure methodological transparency and rigor. Collectively, these strategies uphold the trustworthiness of the qualitative findings, forming a robust foundation for the subsequent quantitative phase.

Phase II will focus on developing and validating a measurement instrument grounded in findings from phase I, aligned with the principles of the PRISMA model. Validation will involve both qualitative methods (expert review) and quantitative techniques (statistical analysis) to ensure reliability, construct validity, and cultural relevance.

In phase III, a cross-sectional survey will be conducted in multiple urban regions using stratified sampling to capture demographic and geographic diversity. The newly developed instrument will be deployed to reduce measurement bias and enhance consistency. Data collection strategies will include trained on-site investigators, real-time supervision, periodic field audits, and quality checks of completed surveys to promote reliability and minimize research bias. Follow-up reminders and incentives will be used to maintain high response rates. Advanced statistical techniques will analyze relationships between key variables while controlling for potential confounders, generating findings that are both reliable and generalizable, with practical implications for implementing integrated care models.

Moreover, integrating findings across all phases will ensure that the qualitative insights meaningfully inform quantitative measurement. The study is expected to yield actionable recommendations for implementing structured, integrated care that addresses health care disparities and supports sustainable, evidence-based older adult care solutions tailored to the Chinese context.

However, reliance on self-reported data may introduce recall bias, and the cross-sectional design may limit the ability to infer causality or track changes in older adults’ expectations over time. While this design is appropriate for assessing the psychometric properties of the newly developed tool, it constrains the study’s capacity to capture temporal dynamics. Moreover, potential sampling bias may arise from recruitment primarily through community medical centers or CECFs, which may not fully capture the diversity of the broader older adult population, potentially those experiencing greater isolation, different health beliefs, or worse mobility. To mitigate this limitation, recruitment will be expanded to include a wide range of community organizations and senior activity groups. Furthermore, stratified sampling based on key demographic factors will be used to ensure representativeness. This will be checked against official census data if available from the government, or against contemporaneous population studies conducted around the same time period as this study.

The study targets urban-dwelling older adults, which may limit the generalizability of findings to rural or remote populations. However, this choice is intentional, as urban-rural disparities in China remain significant in terms of care infrastructure, service availability, professional workforce, and financial support, such as pension and insurance coverage [101-103]. In addition, findings should be interpreted with caution when applied to rural populations, where resource constraints and limited service networks may pose distinct challenges [104]. For example, urban communities have increasingly developed the structural capacity to support integrated care, exemplified by the government’s initiative to build “15-minute older adult care service circles” that provide accessible services such as meal assistance, rehabilitation, and health monitoring within walking distance [105,106]. Given these conditions, urban settings offer an operationally feasible context for the initial implementation of case-managed integrated care. Insights gained from urban experience can serve as a foundation for future adaptation, helping to identify which components of integrated care are transferable and what modifications are needed to ensure feasibility and equity in less-resourced rural contexts.

While this study highlights the potential of adapting the PRISMA model in urban China, several barriers may constrain its translation into practice. Workforce shortages remain a critical challenge, as the supply of trained case managers and health care professionals is insufficient to meet the growing demand. Fragmented care delivery driven by financial constraints at both the community and system levels may also limit the scalability of new care models. Moreover, policy inertia and the complexity of governance may delay the operationalization of initiatives. Nevertheless, the evidence-based framework from this study could serve as an invaluable guide to policymakers, health care stakeholders, researchers, and entrepreneurs to facilitate the implementation of case-managed integrated care for older adults in China.

In conclusion, this study aims to generate empirical evidence to support the adaptation of the PRISMA model in Chinese communities. While findings may provide actionable insights for policy and practice, their impact will depend on addressing structural barriers such as workforce shortages, financial limitations, and policy inertia. By situating recommendations within these constraints, this study provides both evidence and a realistic roadmap for advancing integrated care in non-Western contexts. To ensure the effective translation of research findings into policy, the reporting of results will consider established guidelines for Patient and Public Involvement in Research, such as the Guidance for Reporting Involvement of Patients and the Public checklist [107]. Additionally, the authors plan to engage key stakeholders and patient research partners to facilitate the future adaptation and implementation of the study findings.

## Appendix

Multimedia Appendix 1 Supplement File 1: Theoretical Model Selection based on the IMPACT Framework.

Multimedia Appendix 2 Supplement File 2: The Checklist of Guidelines for Conducting and Reporting Mixed Research for Counselor Researchers.

Multimedia Appendix 3 Supplement File 3. Open-end Questions and Probes.

## Abbreviations

ADL: activities of daily living
BI: Barthel Index
CCM: chronic care model
CECF: community elderly care facility
ICOPE: Integrated Care for Older People program
PRISMA: Program of Research to Integrate the Services for the Maintenance of Autonomy
