# Supplementary material for: Urban Chinese Community-Dwelling Older Adults’ Expectations Regarding the Delivery of Integrated Care Through Case Managers: Protocol for a Mixed Methods Study
Source: JMIR Res Protoc. 2025 Nov 7;14:e71394. doi: 10.2196/71394 (PMC12639349; doi:10.2196/71394)
Supplement: Multimedia Appendix 1 [file resprot_v14i1e71394_app1.docx]

**Supplement File 1: Theoretical Model Selection based on the IMPACT Framework**

| **IMPACT Dimension** | **Chronic Care Model (CCM)** | **Kaiser Permanente Model** | **PRISMA Model** |
| --- | --- | --- | --- |
| **Interestingness** | Focuses on self-management and proactive care [1]. | Integrates funding and service delivery, but lacks exploration in fragmented or public systems, such as China’s [2]. | 1. Introduces case management and cross-sector collaboration in public systems [3]. 2. Aligns with China's evolving long-term care needs and offers novel insights [4, 5]. |
| **Matching** | 1. Designed for integrated clinical settings. 2. Requires substantial system-level change, making alignment with China's decentralized community-based care difficult [1, 6]. | 1. Better suited for vertically integrated private systems. 2. More suitable for micro-level reforms [7].  3. Poorly aligned with China’s public sector-dominant structure [8]. | 1. Fits with the Social Ecological Model and community-level services [9]. 2. Matches well with China’s multilevel, government-supported elderly care ecosystem and emphasizes essential components like case managers, community service centers, voluntary agencies, medical services, and long-term care institutions that are more adaptable to China’s context [10]. |
| **Parsimony** | 1. Contains multiple interdependent components. 2. Requires broad institutional restructuring. | 1. Highly integrated components simplify system-level management. 2. Limited flexibility due to tight coupling. | 1. Focused on essential coordination and case management. 2. Flexible and adaptable across diverse contexts. |
| **Applicability** | 1. Moderately applicable. 2. Implementation in China is complex due to uneven development and limited resources across regions [6]. | 1. Low transferability. 2. Relies on a unified and flexible private system, which contrasts with China’s public-sector-dominated system [8]. | 1. Highly applicable. 2. Supports government-led, public systems [11]. |
| **Conceptual Rigor** | Strong conceptual foundation but less suited to China’s fragmented care system [12]. | Clear structure but limited engagement with the complexity of community-based, cross-sector complexity [7, 13]. | Conceptually rigorous with strong alignment to universal healthcare systems and policy priorities in China [14-17]. |
| **Testability** | Difficult to empirically test in decentralized settings. | Empirically supported in closed systems but lacks external validity for public-sector or universal healthcare systems. | 1. High testability. 2. Core elements are measurable and operationalizable (e.g., needs assessment, level of expectation of case management). 3. Adaptable for scalable tool development. |

References

1. Brettel JH, Manuwald U, Hornstein H, Kugler J, Rothe U: **Chronic‐Care‐Management Programs for Multimorbid Patients with Diabetes in Europe: A Scoping Review with the Aim to Identify the Best Practice**. *Journal of diabetes research* 2021, **2021**(1):6657718.

2. Rompen L, de Vries NM, Munneke M, Neff C, Sachs T, Cedrone S, Cheves J, Bloem BR: **Introduction of network-based healthcare at Kaiser Permanente**. *Journal of Parkinson's disease* 2020, **10**(1):207-212.

3. Hébert R, Durand PJ, Dubuc N, Tourigny A, Group P: **PRISMA: a new model of integrated service delivery for the frail older people in Canada**. *International journal of integrated care* 2003, **3**.

4. Chen X, Giles J, Yao Y, Yip W, Meng Q, Berkman L, Chen H, Chen X, Feng J, Feng Z: **The path to healthy ageing in China: a Peking University–Lancet Commission**. *The Lancet* 2022, **400**(10367):1967-2006.

5. Hu Z, Sun X: **System construction and capacity improvement of home and community elderly care services (CN)**. *Journal of Group Talk* 2023(04):18-21.

6. Zheng L, Zhang L, Chen K, He Q: **Unmasking unexpected health care inequalities in China using urban big data: Service-rich and service-poor communities**. *PLoS One* 2022, **17**(2):e0263577.

7. Li N, Dong Y, Zhang G: **County-Level Integrated Healthcare Practice in China: A Kaiser Permanente-Inspired Approach**. *International Journal of Integrated Care* 2024, **24**(4):18.

8. Moe J, Chen S, Taylor A: **Initial Findings in a Landscaping Study of Healthcare Delivery Innovation in China**. *IPIHD (International Partnership for Innovative Healthcare Delivery) research Report* 2014:14-01.

9. McLeroy KR, Bibeau D, Steckler A, Glanz K: **An ecological perspective on health promotion programs**. *Health education quarterly* 1988, **15**(4):351-377.

10. Feng Z, Glinskaya E, Chen H, Gong S, Qiu Y, Xu J, Yip W: **Long-term care system for older adults in China: policy landscape, challenges, and future prospects**. *The Lancet* 2020, **396**(10259):1362-1372.

11. Li Y, Luo L, Dong H: **Delivering Integrated Community Care for the Elderly: A Qualitative Case Study in Southern China**. *International Journal of Environmental Research and Public Health* 2024, **21**(6):680.

12. Zhu X, Chen AY: **Enlightenment of foreign classical chronic disease management modes on the management of chronic diseases in China (CN)**. *Chinese General Practice* 2023, **26**(01):21.

13. Qiuyi Y, Kumar SSaPA: **Analyzing a New Data-centric Medical Service Model: The Application of Smart Hospitals in China**. *The reflections of new Egypt's strategy to “East Orientation to China” on the leadership’s political legitimacy in front of the American and Israeli intervention and the future polices in the Middle East*.

14. Ma W, Shen Z: **Impact of community care services on the health of older adults: evidence from China**. *Frontiers in Public Health* 2023, **11**:1160151.

15. Wenyi L: **Involvement of social workers in the community service provision system for the older people in urban China**. *Australian Social Work* 2021, **74**(4):448-463.

16. Ma H, Wang M, Yang B: **Research on urban community elderly care facility based on quality of life by SEM: Cases study of three types of communities in Shenzhen, China**. *Sustainability* 2022, **14**(15):9661.

17. Wu W, Divigalpitiya P: **Availability and Adequacy of Facilities in 15 Minute Community Life Circle Located in Old and New Communities**. *Smart Cities* 2023, **6**(5):2176-2195.
