# Supplementary material for: Urban Chinese Community-Dwelling Older Adults’ Expectations Regarding the Delivery of Integrated Care Through Case Managers: Protocol for a Mixed Methods Study
Source: JMIR Res Protoc. 2025 Nov 7;14:e71394. doi: 10.2196/71394 (PMC12639349; doi:10.2196/71394)
Supplement: Multimedia Appendix 2 [file resprot_v14i1e71394_app2.docx]

**Supplement File 2: The Checklist of Guidelines for Conducting and Reporting Mixed Research for Counselor Researchers**

| **Sections** | **Checklist Items** | **Pages** |
| --- | --- | --- |
| **1. Research Formulation** | 1.1.1. Treat each relevant article as data that generate both qualitative (e.g., qualitative findings, literature review of source article, source article author’s conclusion) and quantitative (e.g., *p* values, effect sizes, sample size score reliability, quantitative results) information that yield a mixed research synthesis. |  |
|  | 1.1.2. Subject each document selected as part of the literature review to summarization, analysis, evaluation, and synthesis. |  |
|  | 1.1.3. Provide literature reviews that are comprehensive, current, and rigorous; that have been compared and contrasted adequately; and that contain primary sources that are relevant to the research problem under investigation, with clear connections being made between the sources presented and the present study. |  |
|  | 1.1.4. Present clearly the theoretical/conceptual framework. |  |
|  | 1.1.5. Assess the findings stemming from each individual study and the emergent synthesis for trustworthiness, credibility, dependability, legitimation, validity, plausibility, applicability, consistency, neutrality, reliability, objectivity, confirmability, and/or transferability. |  |
|  | 1.1.6. Present the goal of the study (i.e., predict; add to the knowledge base; have a personal, social, institutional, and/or organizational impact; measure change; understand complex phenomena; test new ideas; generate new ideas; inform constituencies; and examine the past). |  |
|  | 1.2.1. Specify the objective(s) of the study (i.e., exploration, description, explanation, prediction, and influence). |  |
|  | 1.3.1. Specify the rationale of the study. |  |
|  | 1.3.2. Specify the rationale for combining qualitative and quantitative approaches (i.e., participant enrichment, instrument fidelity, treatment integrity, and significance enhancement). |  |
|  | 1.4.1. Specify the purpose of the study. |  |
|  | 1.4.2. Specify the purpose for combining qualitative and quantitative approaches (e.g., identify representative sample members, conduct member check, validate individual scores on outcome measures, develop items for an instrument, identify barriers and/or facilitators within intervention condition, evaluate the fidelity of implementing the intervention and how it worked, enhance findings that are not significant, compare results from the quantitative data with the qualitative findings). |  |
|  | 1.5.1. Avoid asking research questions that lend themselves to yes/no responses. |  |
|  | 1.5.2. Present mixed research questions (i.e., questions that embed both a quantitative research question and a qualitative research question within the same question) when possible. |  |
| **2. Research Planning** | 2.1.1. Specify the initial and final sample sizes for all quantitative and qualitative phases of the study. |  |
|  | 2.1.2. Present all sample size considerations made for the quantitative phase(s) (i.e., a priori power) and qualitative phases (e.g.,information-rich cases). |  |
|  | 2.1.3. Present the sampling scheme for both the quantitative and qualitative phases of the study. |  |
|  | 2.1.4. Describe the mixed sampling scheme (i.e., concurrent–identical, concurrent–parallel, concurrent–nested, concurrent–multilevel, sequential–identical, sequential–parallel, sequential–nested, and sequential–multilevel). |  |
|  | 2.1.5. Clarify the type of generalization to be made (i.e., statistical generalization, analytic generalization, and case-to-case transfer) and link it to the selected sampling design, sampling scheme, and sample size(s). |  |
|  | 2.2.1. Outline the mixed research design. |  |
|  | 2.2.2. Specify the quantitative research design (i.e., historical, descriptive, correlational, causal–comparative/quasi-experimental, and experimental). |  |
|  | 2.2.3. Specify the qualitative research design (e.g., biography, ethnographic, auto-ethnography, oral history, phenomenological, case study, grounded theory). |  |
| **3. Research Implementation** | 3.1.1. Outline the mixed data collection strategy. |  |
|  | 3.1.2. Present information about all quantitative and qualitative instruments and the process of administration. |  |
|  | 3.2.1. Outline the mixed data collection strategy (i.e., data reduction, data display, data transformation, data correlation, data consolidation, data comparison, and data integration). |  |
|  | 3.2.2. Provide relevant descriptive and inferential statistics for each statistical analysis. |  |
|  | 3.2.3. Discuss the extent to which the assumptions (e.g., normality, independence, equality of variances) that underlie the analyses were met, as well as any observations that might have distorted the findings (e.g., missing data, outliers). |  |
|  | 3.2.4. Specify the statistical software used. |  |
|  | 3.2.5. Specify where the responsibility or authority for the creation of categories resided (i.e., participants, programs, investigative, literature, or interpretive), what the grounds were on which one could justify the existence of a given set of categories (i.e., external, rational, referential, empirical, technical, or participative), what was the source of the name used to identify a given category (i.e., participants, programs, investigative, literature, or interpretive), and at what point during the research process the categories were specified (i.e., a priori, a posteriori, or iterative). |  |
|  | 3.2.3. Specify the name of the technique used to analyze the qualitative data (e.g., content analysis method of constant comparison, discourse analysis, componential analysis, keywords in context, analytic induction, word count, domain analysis, taxonomic analysis). |  |
|  | 3.2.7. Specify the qualitative software used. |  |
|  | 3.3.1. Discuss the threats to internal validity, external validity, and measurement validity and outline the steps taken to address each of these threats to internal validity, external validity, and measurement validity. |  |
|  | 3.3.2. Discuss the threats to trustworthiness, credibility, dependability, authenticity, verification, plausibility, applicability, confirmability, and/or transferability of data and outline all verification procedures used. |  |
|  | 3.3.3. Discuss mixed research legitimation types (i.e., sample integration legitimation, insider–outsider legitimation, weakness minimization legitimation, sequential legitimation, conversion legitimation, paradigmatic mixing legitimation, commensurability legitimation, multiple validities legitimation, and political legitimation). |  |
|  | 3.4.1. Interpret relevant types of significance of the quantitative findings (i.e., statistical significance, practical significance, clinical significance, and economic significance). |  |
|  | 3.4.2. Conduct post hoc power analysis for all statistically nonsignificant findings. |  |
|  | 3.4.3. Interpret the significance (i.e., meaning) of qualitative findings. |  |
|  | 3.4.4. Discuss criteria for evaluating findings in mixed research studies (e.g., within-design consistency, conceptual consistency, interpretive agreement, interpretive distinctiveness, design suitability, design fidelity, analytic adequacy, interpretive consistency, theoretical  consistency, integrative efficacy). |  |
|  | 3.5.1. Describe all steps of the mixed research process. |  |
|  | 3.5.2. Describe the context in which the mixed research study took place. |  |
|  | 3.5.3. Ensure that the mixed research report is accurate and complete; does not distort differences within and among individuals and groups; is free from plagiarism or misrepresentation of the ideas and conceptualizations of other scholars; and contains findings that are adequately accessible for reanalysis, further analysis, verification, or replication. |  |
|  | 3.5.4. Present all ethical considerations that were addressed in the study (e.g., informed consent, confidentiality, incentives, funding sources, potential conflicts of interest, biases). |  |
|  | 3.5.5. Specify study approval in accordance with an institutional review board either in the report or in the cover letter submitted to the editor. |  |
|  | 3.5.3. Present recommendations for future research that culminate in a validation, replication, or extension of the underlying study. |  |
