# Supplementary material for: Urban Chinese Community-Dwelling Older Adults’ Expectations Regarding the Delivery of Integrated Care Through Case Managers: Protocol for a Mixed Methods Study
Source: JMIR Res Protoc. 2025 Nov 7;14:e71394. doi: 10.2196/71394 (PMC12639349; doi:10.2196/71394)
Supplement: Multimedia Appendix 3 [file resprot_v14i1e71394_app3.docx]

**Supplement File 3. Open-end Questions and Probes**

| **Sample of interview questions** |
| --- |
| **To older adults:**  ► Do you accept the PRISMA integrated care model?  ► What do you think about the PRISMA integrated care model?  ► Tell us about your experiences with your current medication?  ► What are your unmet needs for accessing healthcare?  ► What sources do you use for healthcare?  ► How do you obtain resources for healthcare?  ► Who will help you when visiting a hospital or clinic?  ► What difficulties do you face when you going to a hospital or clinic?  ► Who will make decisions about your healthcare and how?  ► How do you make decisions about your healthcare or medication?  ► Do you expect assistance from a case manager?  ► What do you expect from case managers?  **To older adults’ children and health professionals**  ► Do you accept the PRISMA integrated care model?  ► What are your opinions on the PRISMA integrated care model?  ► Share with us the challenges of your older parents (or patients) on their current medication  ► What are the unmet healthcare needs of your older parents (or patients) based on your observations?  ► What sources do your older parents (or patients) use for healthcare?  ► How do your older parents (or patients) obtain resources for healthcare?  ► Who assists your older parents (or patients) when they visit a hospital or clinic?  ► What difficulties do your older parents (or patients) facing when going to a hospital or clinic?  ► Who makes decisions for the healthcare of your older parents (or patients) and how are these decisions made?  ► How do your older parents (or patients) make decisions regarding their healthcare and medication?  ► Do you expect assistance from a case manager support your family (or work)?  ► What are your expectations from case managers?  **Probes**  ► Could you provide more details about this?  ► What is the intended meaning of that?  ► Is there anything else you would like to add or address? |
